# Draft Genome Sequence of *Providencia stuartii* PS71, a Multidrug-Resistant Strain Associated with Nosocomial Infections in Greece

**DOI:** 10.1128/genomeA.00056-17

**Published:** 2017-03-23

**Authors:** A. Liakopoulos, O. Oikonomou, D. W. Wareham

**Affiliations:** a Department of Bacteriology and Epidemiology, CVI of Wageningen University, Lelystad, The Netherlands; b Antimicrobial Research Group, Blizard Institute, Barts & the London School of Medicine and Dentistry, Queen Mary University of London, London, United Kingdom; c Division of Infection, Barts Healthcare NHS Trust, London, United Kingdom

## Abstract

*Providencia stuartii* is frequently associated with nosocomial outbreaks and displays intrinsic resistance to many commonly used antimicrobials. We report here the draft genome sequence of a *P. stuartii* strain carrying acquired resistance genes conferring panresistance to cephalosporins (*bla*_SHV-5_ and *bla*_VEB-1_), carbapenems (*bla*_VIM-1_), and aminoglycosides (*rmtB*) involved in an outbreak in Greek hospitals.

## GENOME ANNOUNCEMENT

*Providencia stuartii* has emerged as an important nosocomial pathogen responsible for urinary tract infections in patients with indwelling urinary catheters, hospital-acquired pneumonia, bloodstream infections, and sepsis (1), with significant impact on patient morbidity, mortality, treatment, and management costs (2).

*P. stuartii* isolate PS71 was recovered in 2013 from the bloodstream of a patient during a cluster of *P. stuartii* infections in a critical care unit in a tertiary-care hospital in Athens, Greece (3). This outbreak-associated strain exhibited resistance to cephalosporins (first, second, third, and fourth generation), β-lactam–inhibitor combinations (amoxicillin-clavulanate and piperacillin-tazobactam), carbapenems (ertapenem and imipenem), aminoglycosides, fosfomycin, polymyxins (colistin and polymyxin B), quinolones, and tigecycline (3).

Genomic DNA was extracted and subjected to whole-genome sequencing with the Illumina MiSeq platform (Illumina, Inc., San Diego, CA), producing 2 × 250-bp paired-end reads, generating a total of 843,412 reads with a 475 bp average length. The Trimmomatic algorithm (version 0.36) (4) was used to trim all the generated reads and their quality assessed with in-house scripts using BedTools (version 2.25.0) (5), BWA-mem (version 2) (6), and SAMtools (version 1.3.1) (7) algorithms. High-quality filtered reads were subsequently assembled *de novo* using SPAdes algorithm (version 3.7.1) (8), resulting in 79 scaffolds with an *N*_50_ of 455,952 bp. The sequence coverage of the *de novo* assemblies was approximately 83 reads per assembled base. The draft genome sequence of PS71 consists of 4,411,042 bp with 41.75% average G+C content.

Provisional annotation carried out using the Prokka algorithm (version 1.11) (9) identified at least 4,026 coding sequences (CDSs), including 76 tRNAs and eight rRNAs. The assembled genome was used to predict the presence of acquired antibiotic resistance genes using ResFinder (10) and confirmed the presence of 24 genes conferring resistance to aminoglycosides [*aadA1*, *aadA2*, *aadB*, *aac(6′)-Ia*, *aph(3′)-Ia*, *strA*, *strB*, and *rmtB*], β-lactams (*bla*_TEM-1b_, *bla*_OXA-10_, *bla*_SHV-5_, *bla*_VEB-1_, and *bla*_VIM-1_), macrolides, lincosamides, and streptogramin B [*mph*(*A*)] and phenicols (*cmlA1* and *catA3*), rifampin (*arr-2*), sulfonamides (*sul1* and *sul2*), tetracyclines [*tet*(A), *tet*(B), and *tet*(G)], and trimethoprim (*dfrA1* and *dfrA12*). The presence of multidrug efflux pump-mediated resistance mechanisms was also inferred from the genome (i.e., *acrB*, *acrR*, *cmeB*, *mtrF, mfs*, *macA*, *macB*, and *tolC*), as well as resistance to toxic compounds, including arsenic (*arsA*, *arsB*, *arsC*, *arsD*, and *arsR*), cadmium, cobalt and zinc (*czcB*, *czcD*, *trcD*, and *trMer*), and mercury (*merA*, *merC*, *merD*, *merE*, and *merR*). PlasmidFinder (11) indicated that PS71 contains plasmids with ColE, A/C, and R replicon types, with the latter located on a multireplicon plasmid (3).

There are increasing reports of multiresistant strains of* Providencia* in the Mediterranean, and enhanced surveillance is needed to identify and track the epidemiology of resistant strains, their impact on human health, and the molecular epidemiology. This is the first draft genome sequence of such a multiresistant *P. stuartii* strain associated with a nosocomial outbreak in Greece.

### Accession number(s).

The draft genome sequence of *P. stuartii* PS71 has been deposited in the DDBJ/EMBL/GenBank databases under the accession number MSAA00000000. The version described in this paper is the first version, MSAA01000000.

## References

[1] Armbruster CE, Smith SN, Yep A, Mobley HL 2014 Increased incidence of urolithiasis and bacteremia during *Proteus mirabilis* and *Providencia stuartii* coinfection due to synergistic induction of urease activity. J Infect Dis 209:1524–1532. doi:10.1093/infdis/jit663. 24280366 PMC3997575

[2] Zavascki AP, Carvalhaes CG, da Silva GL, Tavares Soares SP, de Alcântara LR, Elias LS, Sandri AM, Gales AC 2012 Outbreak of carbapenem-resistant *Providencia stuartii* in an intensive care unit. Infect Control Hosp Epidemiol 33:627–630. doi:10.1086/665730. 22561721

[3] Oikonomou O, Liakopoulos A, Phee LM, Betts J, Mevius D, Wareham DW 2016 *Providencia stuartii* isolates from Greece: co-carriage of cephalosporin (*bla*_SHV-5_, *bla*_VEB-1_), carbapenem (*bla*_VIM-1_), and aminoglycoside (*rmtB*) resistance determinants by a multidrug-resistant outbreak clone. Microb Drug Resist 22:379–386. doi:10.1089/mdr.2015.0215. 27380549

[4] Bolger AM, Lohse M, Usadel B 2014 Trimmomatic: a flexible trimmer for Illumina sequence data. Bioinformatics 30:2114–2120. doi:10.1093/bioinformatics/btu170. 24695404 PMC4103590

[5] Quinlan AR, Hall IM 2010 BEDTools: a flexible suite of utilities for comparing genomic features. Bioinformatics 26:841–842. doi:10.1093/bioinformatics/btq033. 20110278 PMC2832824

[6] Li H, Durbin R 2009 Fast and accurate short read alignment with Burrows-Wheeler transform. Bioinformatics 25:1754–1760. doi:10.1093/bioinformatics/btp324. 19451168 PMC2705234

[7] Li H, Handsaker B, Wysoker A, Fennell T, Ruan J, Homer N, Marth G, Abecasis G, Durbin R, 1000 Genome Project Data Processing Subgroup 2009 The sequence alignment/map format and SAMtools. Bioinformatics 25:2078–2079. doi:10.1093/bioinformatics/btp352. 19505943 PMC2723002

[8] Bankevich A, Nurk S, Antipov D, Gurevich AA, Dvorkin M, Kulikov AS, Lesin VM, Nikolenko SI, Pham S, Prjibelski AD, Pyshkin AV, Sirotkin AV, Vyahhi N, Tesler G, Alekseyev MA, Pevzner PA 2012 SPAdes: a new genome assembly algorithm and its applications to single-cell sequencing. J Comput Biol 19:455–477. doi:10.1089/cmb.2012.0021. 22506599 PMC3342519

[9] Seemann T 2014 Prokka: rapid prokaryotic genome annotation. Bioinformatics 30:2068–2069. doi:10.1093/bioinformatics/btu153. 24642063

[10] Zankari E, Hasman H, Cosentino S, Vestergaard M, Rasmussen S, Lund O, Aarestrup FM, Larsen MV 2012 Identification of acquired antimicrobial resistance genes. J Antimicrob Chemother 67:2640–2644. doi:10.1093/jac/dks261. 22782487 PMC3468078

[11] Carattoli A, Zankari E, García-Fernández A, Voldby Larsen M, Lund O, Villa L, Møller Aarestrup F, Hasman H 2014 *In silico* detection and typing of plasmids using PlasmidFinder and plasmid multilocus sequence typing. Antimicrob Agents Chemother 58:3895–3903. doi:10.1128/AAC.02412-14. 24777092 PMC4068535

